# A peculiar new species of gall-inducing, clearwing moth (Lepidoptera, Sesiidae) associated with *Cayaponia* in the Atlantic Forest

**DOI:** 10.3897/zookeys.866.34202

**Published:** 2019-07-24

**Authors:** Gilson R.P. Moreira, Oleg G. Gorbunov, Júlia Fochezato, Gislene L. Gonçalves

**Affiliations:** 1 Departamento de Zoologia, Instituto de Biociências, Universidade Federal do Rio Grande do Sul, Av. Bento Gonçalves 9500, 91501-970 Porto Alegre, RS, Brazil; 2 The A.N. Severtsov Institute of Ecology and Evolution, Russian Academy of Sciences, Leninsky Prospect, 33, Moscow 119071, Russia; 3 PPG Biologia Animal, Departamento de Zoologia, Instituto de Biociências, Universidade Federal do Rio Grande do Sul, Av. Bento Gonçalves 9500, 91501-970 Porto Alegre, RS, Brazil; 4 PPG Genética e Biologia Molecular, Departamento de Genética, Instituto de Biociências, Universidade Federal do Rio Grande do Sul, Av. Bento Gonçalves 9500, 91501-970 Porto Alegre, RS, Brazil; 5 Departamento de Recursos Ambientales, Facultad de Ciencias Agronómicas, Universidad de Tarapacá, Casilla 6-D, Arica, Chile

**Keywords:** Cucurbitaceae, insect galls, *
Melittina
*, *
Neosphecia cecidogena
*, Neotropical region, *
Premelittia
*, sesiid moths, taxonomy

## Abstract

Larvae of most clearwing moths (Lepidoptera, Sesiidae) are endophagous borers of many angiosperms, including their fruits, stems, and roots. Their localized feeding may lead to swellings on those plant parts, but whether the structures produced should be considered true galls is still controversial. In this study we describe a peculiar sesiid moth, *Neospheciacecidogena***sp. nov.** whose larvae induce unusual, external galls on *Cayponiapilosa* (Vell.) Cogn. (Cucurbitaceae) in the Atlantic Forest of southernmost Brazil. The adults, egg, larva, pupa and the gall are described and illustrated based on light and scanning electron microscopy. Galls are cylindrical and unilocular; they are induced individually on axillary buds of the *C.pilosa* stem. Unlike larvae of other sesiids, those of *N.cecidogena***sp. nov.** lack abdominal pseudopodia, and show reduced stemata and chaetotaxy. Pupation occurs inside the gall, after having overwintered in the last larval instar. A maximum likelihood tree constructed based on mitochondrial DNA (COI) sequences showed that *N.cecidogena***sp. nov.** is monophyletic and has an average distance of 13% to species of *Melittia*. The genera *Neosphecia* Le Cerf, 1916 stat. rev., *Premelittia* Le Cerf, 1916 stat rev., and *Melittina* Le Cerf, 1917 stat. rev. are restored from synonyms of *Melittia* Hübner, 1819 [“1816”].

## Introduction

The Sesiidae is a worldwide family with ca 160 valid genera and 1,452 species. Of these, 268 species are found in the Neotropical region (Pühringer and Kallies 2004, 2017) and 119 have been recorded in Brazil (Casagrande et al. 2018). These diurnal micromoths are well known by their morphological and behavioral modifications that have been associated with Batesian mimicry, particularly regarding bees and wasps (Duckworth and Eichlin 1974; Skowron et al. 2015). In the last decades, many of their species have been found by attracting the adults with the use of synthetic sex pheromones (Duckworth and Eichlin 1977b; Brown and Mizell 1993; Špatenka et al. 1999; Arita and Gorbunov 2000; Arita et al. 2009; Skowron et al. 2015; Gorbunov 2018).

In contrast to the adults, however, their immature stages are poorly known, especially in the tropical regions where the host plants for only a small proportion species have been documented (Heppner and Duckworth 1981; Eichlin 2003). The family is known to feed upon a wide variety of shrubs, trees, vines, and herbaceous plants (Heppner and Duckworth 1981; Brown and Mizell 1993; Špatenka et al. 1999). Most sesiid larvae are endophagous borers, and many are highly specific regarding host-plant use. They may be associated with various plant organs, including fruit (Harms and Aiello 1995; Lopes et al. 2003; Puchi 2005), trunks/stems (Bruch 1941; Eichlin and Passoa 1983; Brown and Mizell 1993; Eichlin 1995, 2003), and roots (e.g. Lima 1945; Gorbunov 2015, 2019). Their feeding may lead occasionally to localized swellings on plants, but whether these structures should be considered true galls has been controversial (Eichlin 1995; Hanson et al. 2014). As far we know, none has been associated with the induction of external galls yet.

This study concerns a new species of clearwing moth that induces conspicuous, external galls on the stem of a vine, *Cayaponiapilosa* (Vell.) Cogn. (Cucurbitaceae) in the Atlantic Forest of southernmost Brazil. A preliminary comparison of genitalia structures suggested that it belongs to a genus closely related to *Melittia* Hübner, 1819 [“1816”], but does not conform to any species within it. We describe and illustrate the gall, the immature stages and adults of this moth under both light and scanning electron microscopy, and provide information on its natural history. By conducting a phylogenetic analysis of mitochondrial DNA (COI) sequences using representative members of Melittini (*Melittia*), we provide further support for the proposition of the new taxon, as well as its genus validation.

After careful examination of the habitus we came to the conclusion that this species is congeneric with *Neospheciacombusta* Le Cerf, 1916, which is the type species of the monotypic genus *Neosphecia* Le Cerf, 1916. This genus was formally synonymized with *Melittia* by Duckworth and Eichlin (1977a), but without concrete arguments. Also in this work, by the same reason, we formally restore this and two other names from synonymy with *Melittia* Hübner, 1819 [“1816”]: *Premelittia* Le Cerf, 1916, stat. rev., *Neosphecia* Le Cerf, 1916, stat. rev., and *Melittina* Le Cerf, 1917, stat. rev.

## Material and methods

All specimens used in this study were either dissected or reared from galls in small plastic vials, which were maintained under controlled conditions (14 h light/10 h dark; 25 ± 2 °C) in the Laboratório de Morfologia e Comportamento de Insetos (LMCI), Departamento de Zoologia, Universidade Federal do Rio Grande do Sul (UFRGS). Galls were collected from *C.pilosa* during the autumn seasons of 2014–2018 in the Centro de Pesquisas e Conservação da Natureza (CPCN Pró-Mata/PUCRS; 29°28'36"S, 50°10'01"W), 900 m elevation, São Francisco de Paula Municipality, Rio Grande do Sul State, Brazil. Pupae were obtained later (September) by dissecting some galls under a stereomicroscope in the laboratory. Adults were pin-mounted and dried. Chorionated eggs were obtained by dissection of females used in genitalia preparations. Immature stages were fixed in Dietrich’s fluid and preserved in 75% ethanol. Additional larvae used for DNA extraction were preserved in 100% ethanol at −20 °C.

For descriptions of genital morphology, larvae were cleared in a 10% potassium hydroxide (KOH) solution, stained with either Eosin or Chlorazol black E and slide-mounted in either glycerin jelly or Canada balsam. Last instar larvae were prepared similarly for description of chaetotaxy. Observations were performed with the aid of a Leica M125 stereomicroscope. Structures selected to be drawn were previously photographed with a Sony Cyber-shot DSC-H10 digital camera attached to the stereomicroscope. Vectorized line drawings were then made with the software Corel Photo-Paint X7, using the corresponding digitalized images as a guide. Additional specimens were used for scanning electron microscope analyses. They were dehydrated in a Bal-tec CPD030 critical-point dryer, mounted with double-sided tape on metal stubs, coated with gold in a Bal-tec SCD050 sputter coater and examined and photographed in a JEOL JSM6060 scanning electron microscope at the Centro de Microscopia Eletrônica (CME) of UFRGS.

### Molecular analysis

DNA was extracted from four larvae (specimens LMCI 263-33A, B, C, and D) of the new taxon using the PureLink Genomic DNA extraction kit (Invitrogen). Extracted DNA was resuspended in 50 mL Tris: EDTA (10 mm Tris-HCl, 1 mm EDTA, pH 5 8.0). Mitochondrial DNA PCR was conducted using primers LCO1490 and HCO2198 (Folmer et al. 1994), which amplified approximately 650 bp of the cytochrome oxidase I gene (COI), a fragment entitled DNA barcode. PCR reactions were conducted using 2 µL of the extracted DNA. The thermal cycler profile for CoI consisted of 35 cycles of 94 °C for 45 s, 48 °C for 45 s and 72 °C for 45 s. Excess dNTPs and primers were removed and the amplified DNA concentrated using exonuclease I and FastAP thermosensitive alkaline phosphatase (Thermo Fisher Scientific, Waltham, USA). Samples were sequenced using BigDye Terminator v. 3.1 Cycle Sequencing kit (Thermo Fisher Scientific) and analyzed in an ABI3730XL automatic sequencer. The new data were deposited in the GenBank and BOLD Systems (http://www.boldsystems.org/) under the project MISA (Table 1). The COI sequences were initially aligned using Clustal W (Thompson et al. 1994) and subsequently refined by eye using CodonCode Aligner (CodonCode Corp, Massachusetts, USA).

**Table 1.** DNA barcode sequences of Sesiidae specimens used to infer the relationship of *Neospheciacecidogena* (BOLD dataset DS-NEOSESII) within the tribe Melittiini.

**Group**	**Genus**	**Species**	**Specimen voucher**	**Accession number**	
				**GenBank**	**BOLD**
Ingroup	* Melittia *	*brabanti* Le Cerf, 1917	NS-RR051	JN304556	LNOUE514-11
		*calabaza* Duckworth & Eichlin, 1973	USNM ENT 00831286	MH592747	LNAUS302-12
		*cucurbitae* (Harris, 1828)	USNM ENT 00831289	MH592773	LNAUS305-12
		*grandis* (Strecker, 1881)	USNM ENT 00831295	MH592813	LNAUS310-12
		*gloriosa* Edwards, 1880	USNM ENT 00831292	MH592712	LNAUS308-12
		*snowii* Edwards, 1882	CSU-CPG-LEP001016	GU685631	ABLCU016-09
	* Neosphecia *	*cecidogena* sp. nov.	LMCI 263-33A	MK210242	MISA037-18
			LMCI 263-33B	MK210243	MISA038-18
			LMCI 263-33C	MK210244	MISA039-18
			LMCI 263-33D	MK210241	MISA040-18
Outgroup	* Carmenta *	*bassiformis* (Walker, 1856)	USNM ENT 00831360	MF124173	LNAUS376-12
	* Synanthedon *	*exitiosa* (Say, 1823)	06-JKA-0240	MH592851	LSEU240-06

The final dataset included 12 specimens: four individuals originally sequenced and six species of *Melittia*, the only representative of Melittini available, chosen based on morphologically established relationships with the new taxon. In addition, two species of Synanthedonini were used as outgroup to Melittini according to relationships proposed based on previous molecular phylogenies (e.g. McKern et al. 2008; Hansen et al. 2012; Lait and Hebert 2018).

The specific status of the new taxon was tested through a COI tree relying on monophyly. Tree reconstruction was based on the maximum likelihood (ML) algorithm performed in PHYML v. 3.0 (Guindon et al. 2010), using 1,000 replicates of heuristic search with random addition of sequences and TBR branch swapping. The substitution model GTR+I+G was selected based on the Akaike Information Criterion run in MEGA v. 7 (Kumar et al. 2016). Monophyly confidence limits were assessed with the bootstrap method at a 50% cutoff after 1000 iterations. Sequence divergences were quantified using the Kimura 2-parameter model in MEGA v. 7.

### Museum collections

Abbreviations of the institutions from which specimens were examined are:

**MCTP** Museu de Ciências e Tecnologia da Pontifícia Universidade Católica do Rio Grande do Sul, Porto Alegre, Rio Grande do Sul, Brazil.

**LMCI** Laboratório de Morfologia e Comportamento de Insetos, Universidade Federal do Rio Grande do Sul, Porto Alegre, Rio Grande do Sul, Brazil.

## Results

### Molecular phylogeny

**Figure 1.**
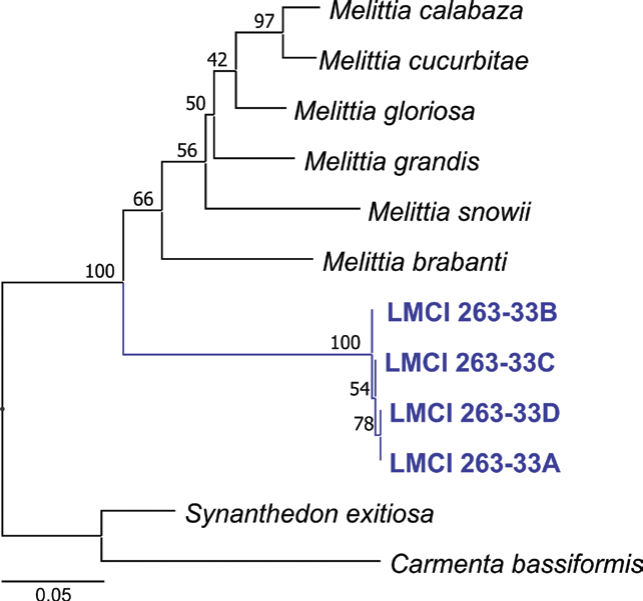
Maximum likelihood consensus tree of *Neospheciacecidogena* (LMCI 263-33) and related Melittiini taxa. The monophyly and relationships were inferred based on 668 base pairs of cytochrome oxidase subunit gene I (COI) sequences (using six species of *Melittia*, one from *Synanthedon* and *Carmenta* from databases; the later genera of the related subfamily Synanthedonini were surveyed as outgroups). Numbers above branches indicate statistical support (bootstrap values).

Sequencing of COI resulted in an average amplicon size of 633 bp. The aligned data matrix including all genera resulted in 668 characters. Of these, 189 (28%) were phylogenetically informative. Maximum likelihood analysis recovered an optimal ML tree = 5897 with nucleotide frequencies of A = 29.1%, C = 16.5%, G = 14.8%, and T = 39%. The four LMCI 263-33 specimens grouped in the COI tree as a single lineage, which was placed close to the genus *Melittia* (Fig. 1). *Melittiagrandis* was the closest taxon (89% similarity) retrieved from online blast tools of Genbank and BOLD. Pairwise K2P distances estimated from the new taxon and *Melittia* ranged from 13% to 16% (*M.grandis* and *M.calabaza*, respectively) (Table 2). Divergence to the outgroup varied from 18% to 22%.

**Table 2.** Genetic distance between *Neospheciacecidogena* and other members of Melittini based on a 668 base pair sequence of the cytochrome oxidase I (COI) gene using the Kimura 2-parameter model. Comparisons with the outgroup Synanthedonini (*Synanthedon* and *Carmenta*) are indicated as well. Intra-group distances are shown in brackets.

	**1**	**2**	**3**	**4**	**5**	**6**	**7**	**8**	**9**
1. *Neospheciacecidogena*	0.00								
2. *Melittiasnowii*	0.14	–							
3. *Melittiabrabanti*	0.15	0.11	–						
4. *Melittiacalabaza*	0.13	0.08	0.11	–					
5. *Melittiagloriosa*	0.13	0.08	0.08	0.05	–				
6. *Melittiagrandis*	0.12	0.09	0.09	0.06	0.06	–			
7. *Melittiacucurbitae*	0.14	0.09	0.10	0.02	0.05	0.06	–		
8. *Synanthedonexitiosa*	0.18	0.16	0.16	0.18	0.15	0.16	0.18	–	
9. *Carmentabassiformis*	0.22	0.21	0.19	0.19	0.19	0.18	0.20	0.12	–

### Taxonomy

#### Melittina

Taxon classificationAnimaliaLepidopteraSesiidae

Le Cerf, 1917 stat. rev.

57b1c0d4-365a-56f2-8bfc-02d20fbb23a9

“Genre Melittina gen. nov.” Le Cerf, 1917: 239. Type species: Melittinanigra Le Cerf, 1917, by original designation.
Dalla Torre and Strand 1925: 136 (as a distinct genus); Zukowsky 1936: 1248 (as a distinct genus); Duckworth and Eichlin 1977a: (as a synonym of Melittia); Heppner and Duckworth 1981: 26 (as a synonym of Melittia); Eichlin and Duckworth 1988: 50 (as a synonym of Melittia); Pühringer and Kallies 2004: 15 (as a synonym of Melittia).

##### Diagnosis

(after Le Cerf 1917: 239; pl. 477, fig. 3933). Head with antenna strongly clavate but without a hook apically, short, slightly shorter than half forewing; vertex with hair-like scales; proboscis well developed, functional. Legs with hind tibia and tarsus with slightly elongated scales. Forewing with transparent areas well developed; veins R_4_ and R_5_ long stalked; hindwing transparent, anal lobe undeveloped. Abdomen smooth scaled with anal tuft poorly developed.

##### Differential diagnosis.

Despite the fact that Le Cerf placed *Melittina* in the subfamily Aegeriinae sensu Le Cerf, 1917 [= Sesiini + Osminiini + Paranthrenini + Synanthedonini], by the venation of the hindwing this genus belongs to the tribe Melittiini. Superficially *Melittina* resembles the Afrotropical genus *Agriomelissa* Meyrick, 1931 (type species: *Agriomelissagypsospora* Meyrick, 1931), but it can be differed by the practically undeveloped hair-like tuft of the hind leg (vs hair-like tuft of the hind leg well-developed in *Agriomelissa*) and undeveloped anal lobe of the hindwing (vs well-developed in the genus compared). *Melittina* can be easily distinguished from *Premelittia* and *Neosphecia* by the well-developed proboscis (proboscis undeveloped in both these compared genera) and shape of the antenna (antenna strongly clavate, short, slightly shorter than a half of forewing in *Melittina*, vs antenna fusiform, slightly longer than middle of forewing in *Premelittia* and *Neosphecia*). From all other genera in Melittiini, including species of New World so-called “Melittia” (Arita and Gorbunov 1996), *Melittina* differs by the shape of the antenna (antenna clavate with a hook apically in compared Melittiini) and by the undeveloped anal lobe of the hindwing (anal lobe of the hindwing well developed in all genera compared Melitiini).

##### Composition.

Presently this genus contains only a single species: *Melittinanigra* Le Cerf, 1916, stat. rev.

##### Remarks.

The genus *Melittina* was described by Le Cerf (1917) based on a single female specimen of the type species, *Melittinanigra* Le Cerf, 1917. Fortunately, the original description is rather complete and supplemented with a fairly accurate figure of the type species, *Melittinanigra* (Le Cerf 1917: 239; pl. 477, fig. 3933). They contain important characters (venation of the hindwing), which clearly put this genus in the tribe Melittiini. As we have mentioned above, this genus was synonymized with *Melittia* by Duckworth and Eichlin (1977a) without concrete arguments. After a careful re-examination of the original description (Le Cerf 1917: 239) and the figure of the type species (Le Cerf 1917: pl. 477, fig. 3933), we conclude that *Melittina* is a distinct genus and restore it from synonymy with *Melittia*. Besides this, we also transfer *Melittianigra* (Le Cerf, 1917) back to its original combination, *Melittinanigra* Le Cerf, 1917.

#### Premelittia

Taxon classificationAnimaliaLepidopteraSesiidae

Le Cerf, 1916 stat. rev.

ceb4b7d7-e285-543d-96af-6e0b664b380e

“Premelittiarufescens ♀ nov. sp.” — Le Cerf 1916: 9. Type species: Premelittiarufescens Le Cerf, 1916, by original designation.
Dalla Torre and Strand 1925: 136 (as a distinct genus); Zukowsky 1936: 1248 (as a distinct genus); Duckworth and Eichlin 1977a: (as a synonym of Melittia); Heppner and Duckworth 1981: 25 (as a synonym of Melittia); Eichlin and Duckworth 1988: 50 (as a synonym of Melittia); Špatenka et al. 1999: 89 (as a synonym of Melittia); Pühringer and Kallies 2004: 15 (as a synonym of Melittia).

##### Diagnosis

(after Le Cerf 1916: pl. 375, fig. 3136, 1917: 234). Head with antenna fusiform without a hook distally, slightly longer than half forewing; vertex smooth scaled; proboscis undeveloped. Legs with hind tibia and tarsus smoothly scaled. Forewing with transparent areas well-developed; veins R_4_ and R_5_ short stalked; hindwing transparent, anal lobe very small. Abdomen smoothly scaled with anal tuft poorly developed.

##### Differential diagnosis.

Without any doubt, by the venation of the hindwing this genus belongs to the tribe Melittiini. By the undeveloped proboscis and fusiform antenna this genus seems to be closely related to the genus *Neosphecia* Le Cerf, 1916, but it can be separated from it by the smoothly scaled vertex (vs vertex covered with hair-like scales in the compared genus) and venation of the forewing (veins R_4_ and R_5_ short stalked in *Premelittia* vs veins R_4_ and R_5_ separate basally in *Neosphecia*). It is easily distinguished from all other Melittiini, including species of New World so-called “Melittia” (Arita and Gorbunov 1996), by the smoothly scaled vertex (vs vertex with hair-like scales in all genera of Melittiini), form of the antenna (antenna clavate with a hooked apex in all other Melittiini), absence of the proboscis (proboscis well developed and functional in all other Melittiini), and form of the hind leg (with a tuft of hair-like scales in all other Melittiini).

##### Composition.

Presently this genus contains the single species: *Premelittiarufescens* Le Cerf, 1916.

##### Remarks.

The generic name *Premelittia* was first introduced in the legends of figures as the binominal “*Premelittiapufescens* nov. sp.” (Le Cerf 1916: 9). The original description of this genus was published a year later (Le Cerf 1917: 234). Unfortunately, it was based on a single female specimen of the type species, *Premelittiapufescens* Le Cerf, 1916, but it is rather complete and contains important characters, which clearly separate this genus from the genus *Melittia*. This genus was also synonymized with *Melittia* by Duckworth and Eichlin (1977a) without concrete arguments. After a careful re-examination of the description (Le Cerf 1917: 234) and the figure of the type species (Le Cerf 1916: pl. 375, fig. 3136) we conclude that *Premelittia* is a distinct genus of the tribe Melittiini and restore it from synonymy with *Melittia*. Besides this, we also transfer *Melittiarufescens* (Le Cerf, 1916) back to its original combination, *Premelittiarufescens* Le Cerf, 1916.

#### Neosphecia

Taxon classificationAnimaliaLepidopteraSesiidae

Le Cerf, 1916 stat. rev.

4277883d-58a8-5d4b-aa39-71e9a3402a78

“Neospheciacombusta ♀ nov. sp.” — Le Cerf 1916: 9. Type species: Neospheciacombusta Le Cerf, 1916, by original designation.
Dalla Torre, Strand 1925: 136 (as a distinct genus); Zukowsky 1936: 1247 (as a distinct genus); Duckworth and Eichlin 1977a (as a synonym of Melittia); Heppner and Duckworth 1981: 26 (as a synonym of Melittia); Eichlin and Duckworth 1988: 50 (as a synonym of Melittia); Pühringer and Kallies 2004: 15 (as a synonym of Melittia).

##### Diagnosis

(after Le Cerf 1916: pl. 375, fig. 3137, 1917: 236). Head relatively broad; antenna fusiform without a hook distally, slightly longer than half forewing; vertex covered with hair-like scales; proboscis undeveloped. Legs with hind tibia and tarsus smoothly scaled. Forewing with transparent areas undeveloped or very small; veins R_4_ and R_5_ separate basally; hindwing transparent, anal lobe undeveloped or extremely small. Abdomen smooth scaled with anal tuft poorly developed.

##### Differential diagnosis.

Like the two previous taxa, on the basis of the venation of the hindwing this genus belongs to the tribe Melittiini. By the fusiform antenna and undeveloped proboscis this genus is closely related to the genus *Premelittia* Le Cerf, 1916, stat. rev., but it can be distinguished the forewing venation (veins R_4_ and R_5_ shortly stalked in *Premelittia* vs veins R_4_ and R_5_ separate basally in *Neosphecia*). *Neosphecia* differs from *Melittina* by the undeveloped proboscis (well developed and functional in the compared genus), shape of the antenna (strongly clavate and short, not reaching the middle of the forewing in *Melittina* vs fusiform, long, slightly longer than half forewing in *Neosphecia*), and the venation of the forewing (veins R_4_ and R_5_ long stalked in *Melittina* vs veins R_4_ and R_5_ separate basally in *Neosphecia*). From all other genera of the Melittiini, including New World so-called “Melittia” (Arita and Gorbunov 1996), it differs by the shape of the antenna (clavate with a hook apically in all other Melittiini vs fusiform in *Neosphecia*), completely undeveloped proboscis (vs well developed in all other Melittiini), and venation of the forewing (veins R_4_ and R_5_ separate basally in *Neosphecia* vs veins R_4_ and R_5_ stalked in all genera of Melittiini).

##### Composition.

Currently, we include two species in this genus: *Neospheciacombusta* Le Cerf, 1916 (type species) and *N.cecidogena* sp. nov.

##### Remarks.

Like the genus *Premelittia* (see above), the genus name *Neosphecia* was firstly introduced in the legends of the figures as the binomen “*Neospheciacombusta* nov. sp.” (Le Cerf 1916: 9). The original description of this genus was published a year later (Le Cerf 1917: 236). Unfortunately, it was based on a single female specimen of the type species, *Neospheciacombusta* Le Cerf, 1916, but it is rather complete and contains important characters, which clearly separate this genus from *Melittia*. This genus was also synonymized with *Melittia* by Duckworth and Eichlin (1977a) without concrete arguments, as with the two previous genera. After careful re-examination of the description (Le Cerf 1917: 236) and the figure of the type species (Le Cerf 1916: pl. 375, fig. 3137) we conclude that *Neosphecia* is a distinct genus of the tribe Melittiini and restore it from synonymy with *Melittia*. Besides this, we also transfer *Mellittiacombusta* (Le Cerf, 1916) back to its original combination, *Neospheciacombusta* Le Cerf, 1916.

#### Neosphecia cecidogena

Taxon classificationAnimaliaLepidopteraSesiidae

Moreira & Gorbunov sp. nov.

351a3a7e-6a57-5a5a-b092-2de77cb8b1cc

http://zoobank.org/116E59F8-44F9-4F1A-9CAD-6C32C068E4E4

Figs 2 , 3 , 4 , 5 , 6 , 7 , 8 , 9

##### Description.

**Figure 2.**
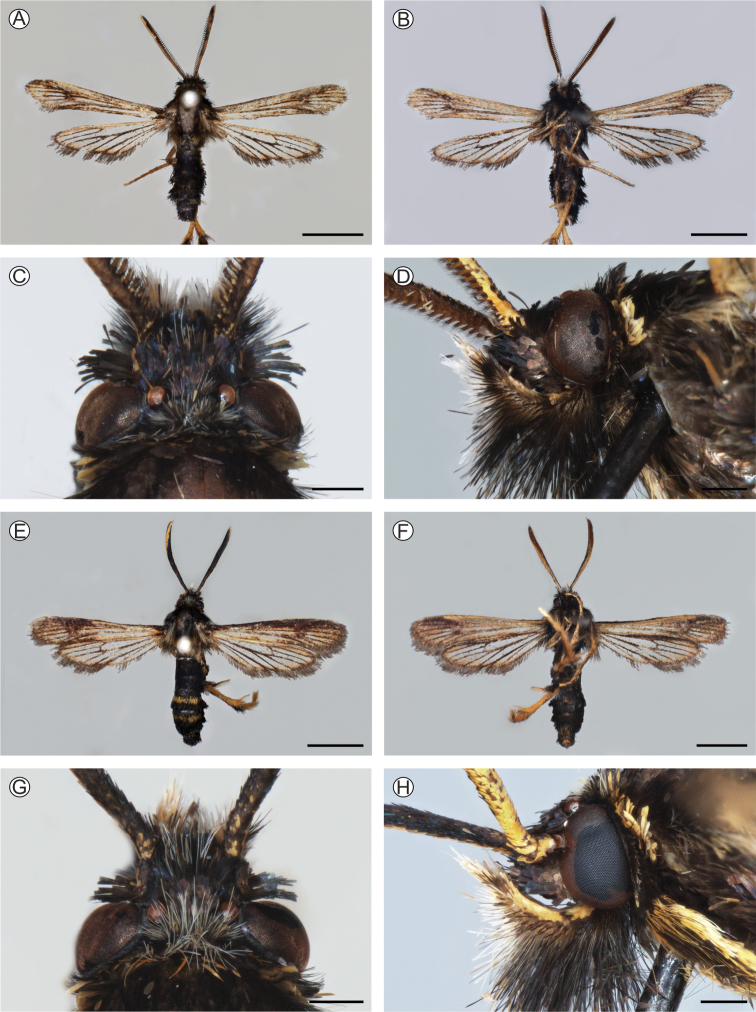
Pinned-dried *Neospheciacecidogena* adults, with corresponding heads in detail **A–D** male (holotype, LMCI 319-83) **E–H** female (paratype, LMCI 319-85); dorsal (**A, C, E, G**), ventral (**B, F**) and lateral (**D, H**) views. Scale bars = 0.5mm (**C, D, G, H**); 4mm (**A, B, E, F**).

**Male** (holotype) (Fig. 2A–D). Alar expanse 23.1 mm; body length 10.8 mm; forewing 10.5 mm; antenna 5.8 mm.

Head with antenna dark brown to black dorso-externally and yellow ventro-externally; scapus yellow and narrowly lined with dark brown dorsally; frons entirely dark brown with purple-blue sheen; vertex black with dark-blue sheen and an admixture of individual white and yellow hair-like scales; proboscis completely undeveloped; labial palpus dark brown to black with an admixture of yellow scales dorsally and white, long, hair-like scales ventrally in distal half; occipital black with a few white scales dorsally.

Thorax with patagia dark brown to black with a small, yellow, transverse spot anterior-ventrally; tegula dark brown to black with yellow, hair-like scales distally; mesothorax entirely dark brown to black; metathorax dark brown to black with two tufts of yellow, hair-like scales laterally; thorax laterally dark gray-brown with bronze-violet sheen. Legs with neck plate dark brown to black; fore coxa dark brown to black with a narrow, yellow exterior margin; hind tibia dark yellow with an admixture of black elongated scales on basal half; spurs dark yellow with golden sheen and a few black scales exterior-basally; hind tarsus dark yellow with a dense admixture of elongated black scales dorso-externally. Forewing: dorsally dark brown with dark-violet sheen and an admixture of individual yellow-orange scales, more dense distally and at anal margin; transparent areas present but very small: anterior and posterior ones very narrow, external one divided into two very short cells; cilia dark brown to black with dark violet-purple sheen. Hindwing transparent; veins broadly covered with dark brown and a few yellow-orange scales; discal spot undeveloped; outer margin about as broad as cilia, dark yellow and narrowly dark brown distally; cilia dark brown with dark violet sheen.

Abdomen including anal tuft black with dark blue sheen and a few yellow scales at base of second tergite.

**Figure 3.**
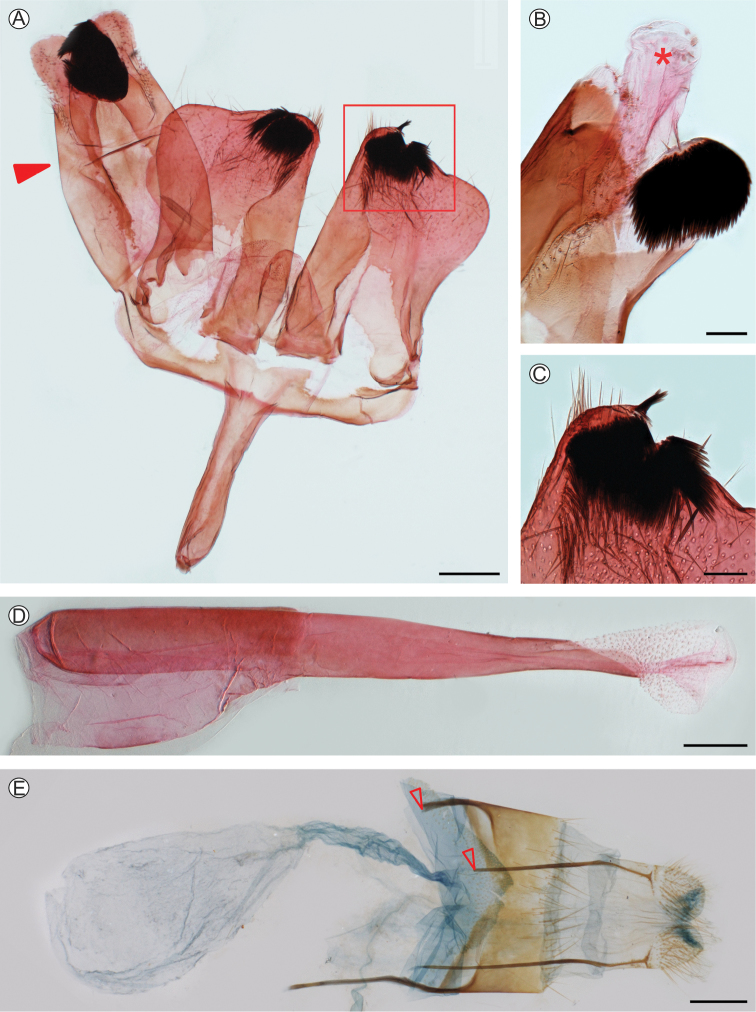
*Neospheciacecidogena* genitalia morphology under light microscopy **A** male (LMCI 319-84), general, ventral view (unrolled preparation, sensu Pitkin 1986; aedeagus omitted) **B** right half of the tegumen-uncus complex, mesal (pointed by closed arrow in **A** asterisk indicates anal tube) **C** distal portion of left valva in detail (enlarged area marked with a rectangle in **A**), ventral **D** aedeagus, lateral **E** female (LMCI 306-19), general, ventral (open arrow points to missing distal portion of the right anterior apophysis, broken off during preparation). Scale bars = 0.1 mm (**B–D**); 0.3 mm (**A, E**).

***Male genitalia*** (Fig. 3A–D). Tegumen-uncus complex relatively broad; uncus bilobed distally, with a relatively large semi-oval plate of strong, short, pointed setae internally on each side distally; gnathos rather small, membranous, with a small, narrow, slightly sclerotized plate medio-basally; valva broad, subrectangular, with dorsal margin concave mesally and rounded distally; distal field of setae not developed; setae of medial field restricted to a path on ventro-distal margin; ventral lobe relatively broad on 2/3 basal section, narrowed distally; saccus narrow, ca 0.7 valva in length; aedeagus tubiform, narrowed distally, ca 1.3× valva length; vesica with numerous minute cornuti.

**Female** (paratype) (Fig. 2E–H). Antenna with more broad yellow stripe ventro-externally; vertex with more numerous white hair-like scales; labial palpus with more numerous yellow scales dorsally; patagia with more yellow scales anteriorly; legs with more numerous yellow scales; both tergites 4 and 5 with a sparse, dark yellow stripe medially. Color patterns otherwise as in male.

***Female genitalia*** (Fig. 3E). Papillae anales membranous, covered with short and a few long setae; eighth tergite relatively broad with relatively long setae distally; posterior apophyses about 1.2× longer than anterior apophyses; ostium bursae opening near posterior margin of sternite seven, slightly funnel-shaped; antrum membranous, narrow and short; ductus bursae narrow, slightly broadened medially, about as long as anterior apophyses; corpus bursae membranous, elongate-ovoid, ca 1.5× as long as anterior apophyses, without signum.

##### Individual variability.

The type series practically invariable in individual size and in the coloration of various parts of the body and wings.

##### Differential diagnosis.

This new species looks like *Melittinanigra* Le Cerf, 1917 (type locality: “Brésil, ex E. Le Moult, Coll. F. Le Cerf”; Le Cerf 1917: 240), from which it can be easily distinguished by the absence of the proboscis (well developed in *M.nigra*) and poorly developed transparent areas of the forewing (large, well developed, external transparent area divided into seven cells between veins R_3_–CuA_2_ in *M.nigra*; compare Fig. 2 with Le Cerf 1917: pl. 477, fig. 3933). From *N.combusta* Le Cerf, 1916 (type locality: “Bolivie, Cochabamba, Yunga del Espiritu-Santo; ex P. Germain (1888–1889)”; Le Cerf 1916: 9) this new species differs by the presence of transparent areas of the forewing (completely opaque in *N.combusta*), by the coloration of the abdomen (dorsally tergite 3 with a narrow yellow stripe anteriorly in *N.combusta*), and by the coloration of the hind tarsus (dark brown to black in the compared species; compare Fig. 2 with Le Cerf 1916: pl. 375, fig. 3137). *Neospheciacecidogena* sp. nov. cannot be confused with any other Melittiini of the Neotropical region by its generic characters.

##### Etymology.

The species name, an adjective, is derived from a composition between the Portuguese “Cecidia” (a gall) and the suffix *gena* (derived from the Latin verb “gigno”, be born). Thus, the epithet refers to the cecidogenous habit of the new described clearwing moth.

##### Material examined.

All specimens were either dissected or reared from galls associated with *Cayaponiapilosa* (Vell.) Cogn. (Cucurbitaceae), from the Centro de Pesquisas e Conservação da Natureza Pró-Mata (CPCN Pró-Mata, 29°28'36"S, 50°10'01"W, São Francisco de Paula Municipality, Rio Grande do Sul State (RS), Brazil; 04–06.IV.2014, G.R.P. Moreira & R. Brito legs., LMCI 263; 21–24.VI.2016, G.R.P. Moreira, R. Brito, J. Fochezato legs, LMCI 306; 28–30.VI.2017, G.R.P. Moreira and J. Fochezato legs., LMCI 319; 01–02.VIII.2017, G.R.P. Moreira and J. Fochezato, LMCI 320; 20–23.III.2018, G.R.P. Moreira, V. Becker, A. Moser, R. Brito & J. Fochezato legs., LMCI 326.

**Type material** (all pinned-dried adults). *Holotype*: ♂ LMCI 319-83; *Paratypes*: 1♂, LMCI 319-84, with genitalia preparation on slide; 1♀, LMCI 263-52, with genitalia preparation on slide; 1♀, LMCI 319-82, donated to MCTP (64103); 1♀, LMCI 319-85.

**Non-type material.** Adults (pinned-dried): 1♂, with genitalia preparation on slide, LMCI 319-81; 1♀, with genitalia preparation on slide, LMCI 306-19. Immature stages (fixed in Dietrich’s fluid and preserved in 70% ethanol): ca 30 eggs, dissected from female during genitalia preparation, LMCI 263-52b; 2 last instar larvae (LMCI 263-49 and 326-148); 2 pupae (LMCI 263-51 and 309-02); 12 dissected, mature galls (LMCI 263-35); 5 empty, senescent galls with pupal exuviae (LMCI 319-86). Also, 6 last instar larvae, preserved in 100% ethanol at −20 °C, used for DNA extraction (4 specimens, LMCI 263-33; 2 specimens, 326-146), and 2 last instar larvae preparations, mounted in Canada balsam on a slide (LMCI 263-42, 43).

##### Description of immature stages.

**Figure 4.**
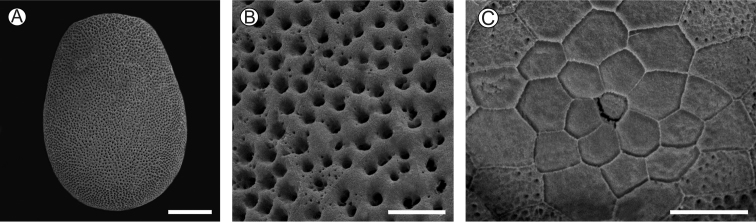
Egg of *Neospheciacecidogena* under scanning electron microscopy **A** general view **B** chorion in detail **C** micropyle area. Scale bars: 100, 15, 20 µm, respectively.

**Eggs** (Fig. 4): light brown, obovoid, with the anterior end slightly flattened; maximum length (average ± standard deviation) = 0.05 ± 0.01 mm, maximum width = 0.39 ± 0.01 mm, *n* = 6. Surface of chorion with faint carenae, delimiting irregular, mostly hexagonal cells and minutely pitted, forming a continuous meshwork-like plastron (sensu Hinton 1981), except for the anterior end where corresponding holes are sparse. Micropylar area on anterior pole, consisting of a subtrapezoidal indentation in the center, which is surrounded by a rosette of about 20 subpentagonal cells that increase in size centrifugally.

**Figure 5.**
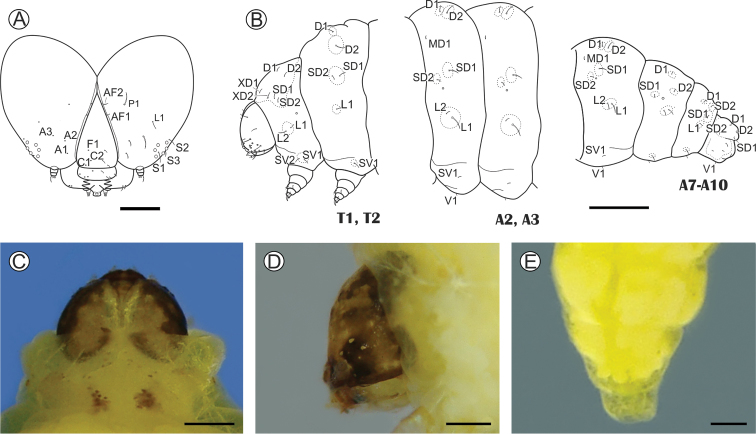
*Neospheciacecidogena* last larval instar under light microscopy **A** cephalic chaetotaxy, frontal view **B** thoracic and abdominal chaetotaxy, lateral **C, D** head in detail, anterior and lateral, respectively. **E** last abdominal segments in detail, dorsal. Scale bars: 200 µm (**A, D**); 0.4 mm (**E**); 0.5 mm (**C**); 1 mm (**B**).

**Figure 6.**
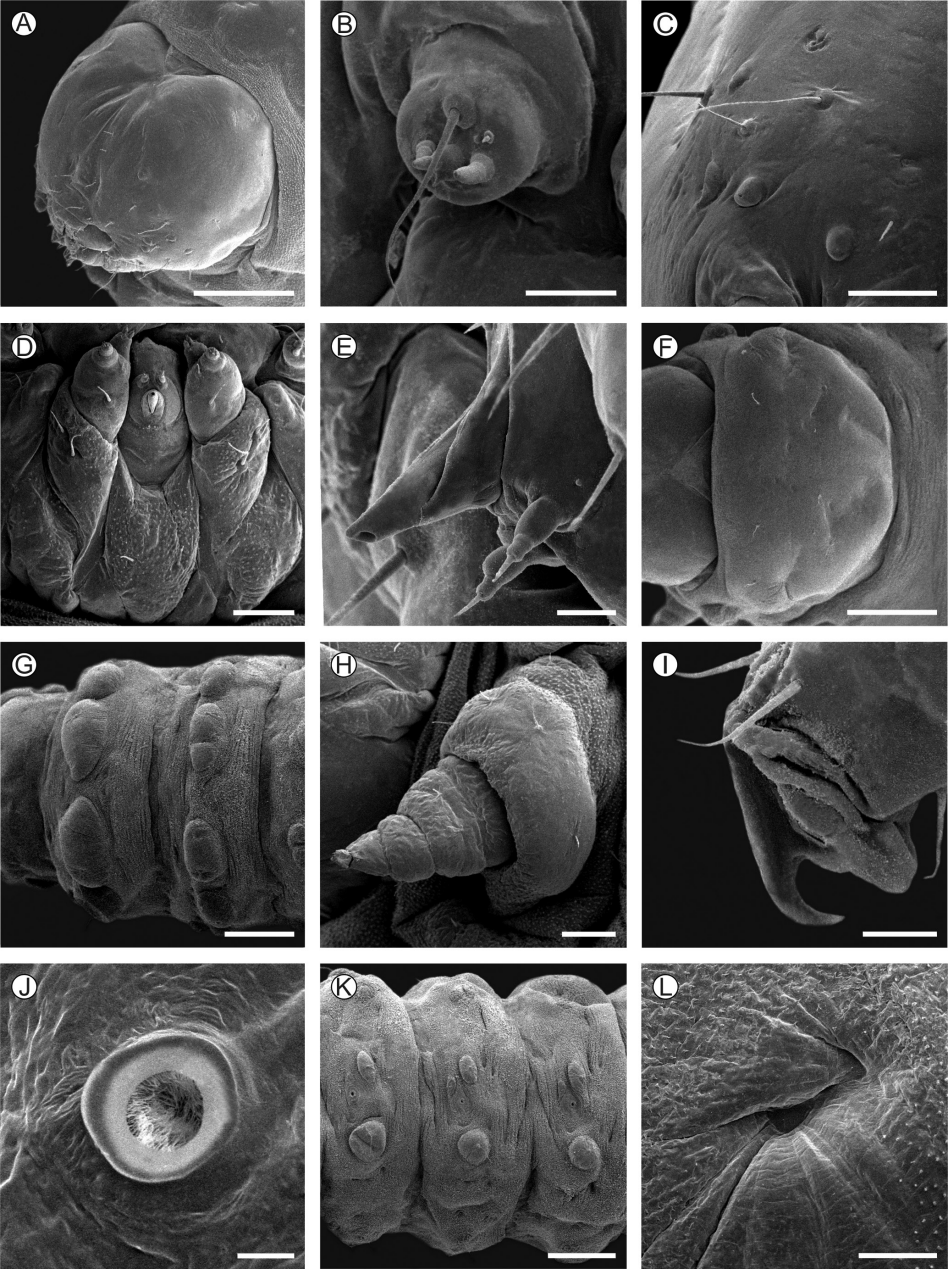
Morphology of *Neospheciacecidogena* last larval instar under scanning electron microscopy **A** head, antero-dorsal view **B** antenna, anterior **C** stemmata, lateral **D** maxillae and labium, ventral **E** spineret, lateral **F** prothoracic shield, dorsal **G** meso- and metathoracic segments, dorsal **H** prothoracic leg, posterior **I** tarsal claw in detail, posterior **J** prothoracic spiracle, lateral **K** second to fourth abdominal segments, lateral **L** abdominal callus in detail, ventral. Scale bars: 20 µm (**E, I, J**); 40 µm (**B**), 100 µm (**C, D, H, L**); 0.5 mm (**A, G, K**).

**Last instar larva** (Figs 5, 6, 9D): head capsule width (average ± standard deviation) = 2.39 ± 0.06 mm; body length = 10.34 ±2.45 mm, *n* = 4. Body light yellow; head tan-brown, with a clearer, dorsal, irregularly shaped area, covering the frontoclypeus, adfrontal area and adjacent portions; this area projects latero-posteriorly, ending close to the posterior margin of the head. Prothoracic shield slightly melanized except for a pair of faint patches formed by pigmented spots, located mesally on posterior margin. Anal plate and prothoracic legs not melanized (Figs 5C, D, 9D). Setae mostly reduced in size, on pinacula (Fig. 6G, K) that are inconspicuous under light microscopy (same color as body) (Fig. 5C–E). Head: wider than long, with lateral margins convex, slightly hypognathus; frontoclypeus subtriangular, higher than wide, extending to three-quarters of epicranial notch; ecdysial line weakly defined, reaching close to epicranial notch and delimiting a narrow adfrontal area (Fig. 5A–D). Six poorly developed, laterally located stemmata (Fig. 6A, C). Antennae (Fig. 6B) two-segmented; basal segment with four sensilla on distal margin, two short and stout, one minute and one long, ca 10× the length of the others; distal segment much thinner and shorter, bearing three short sensilla on distal margin. Labrum slightly bilobed, with three pairs of setae laterally on distal margin, and one pair centrally on proximal base. Mandible well developed, with four cusps along distal margin and two small setae mesally on external surface. Maxilla (Fig. 6D, E) with palpus and galea well developed. Spinneret short, conical (Fig. 6D, E). Labial palpus (Fig. 6E) bisegmented; distal segment thinner and shorter, with well-developed apical seta. Thorax (T) and abdomen (A): integument covered with microtrichia, except on pinacula (Fig. 6G, K, L). Thoracic legs well developed, with stout tarsal claw (Fig. 6H, I). Circular spiracles with slightly elevated peritreme, laterally on T1, A1–8. Abdominal pseudopodia absent, replaced by pairs of ambulatory calli (Fig. 6L) on A3–6 and A10, without crochets.

Chaetotaxy (Fig. 5A, B). Head with F unisetose; C group bisetose; A group trisetose, forming an obtuse triangle with A3 closest to stemmata; AF group bisetose; P unisetose; Md group absent; L unisetose; S trisetose; SS trisetose (not drawn). A3 and P1 about equal in length, longest setae on head. T1 with D group bisetose; XD bisetose; SD bisetose; L bisetose; SV bisetose. T2–3 with D group bisetose; SD unisetose; L1 unisetose; SV unisetose. A1–7 with D group bisetose; MD unisetose; SD bisetose; L bisetose; SV and V unisetose. A8 with D group bisetose; MD unisetose; SD1 unisetose; L bisetose; V unisetose. A9 with D group unisetose, SD bisetose; L unisetose. A10 with D group bisetose; SD bisetose; V unisetose, and three pairs of unnamed setae on lateral of calli.

**Figure 7.**
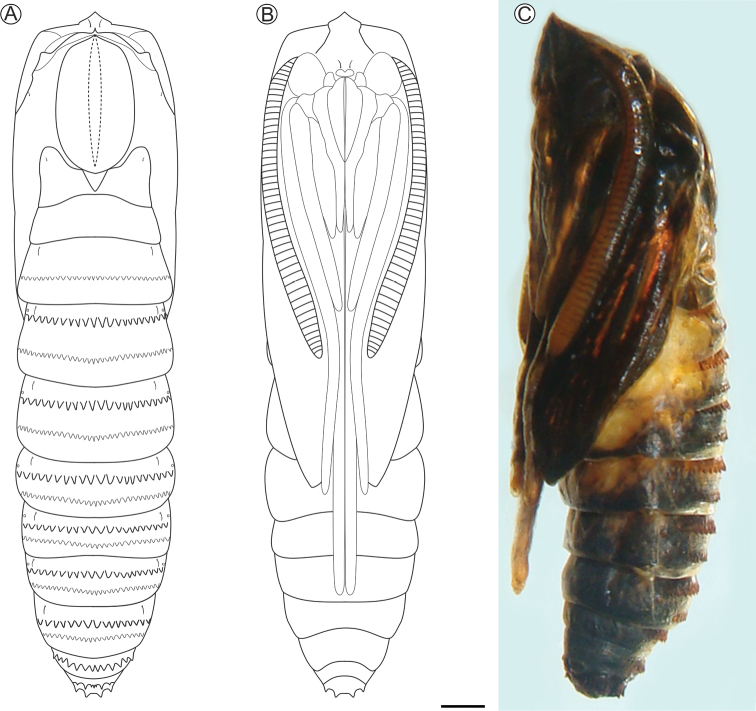
*Neospheciacecidogena* pupa under light microscopy, in dorsal (**A**), ventral (**B**) and lateral (**C**) views. Scale bar:1 mm.

**Figure 8.**
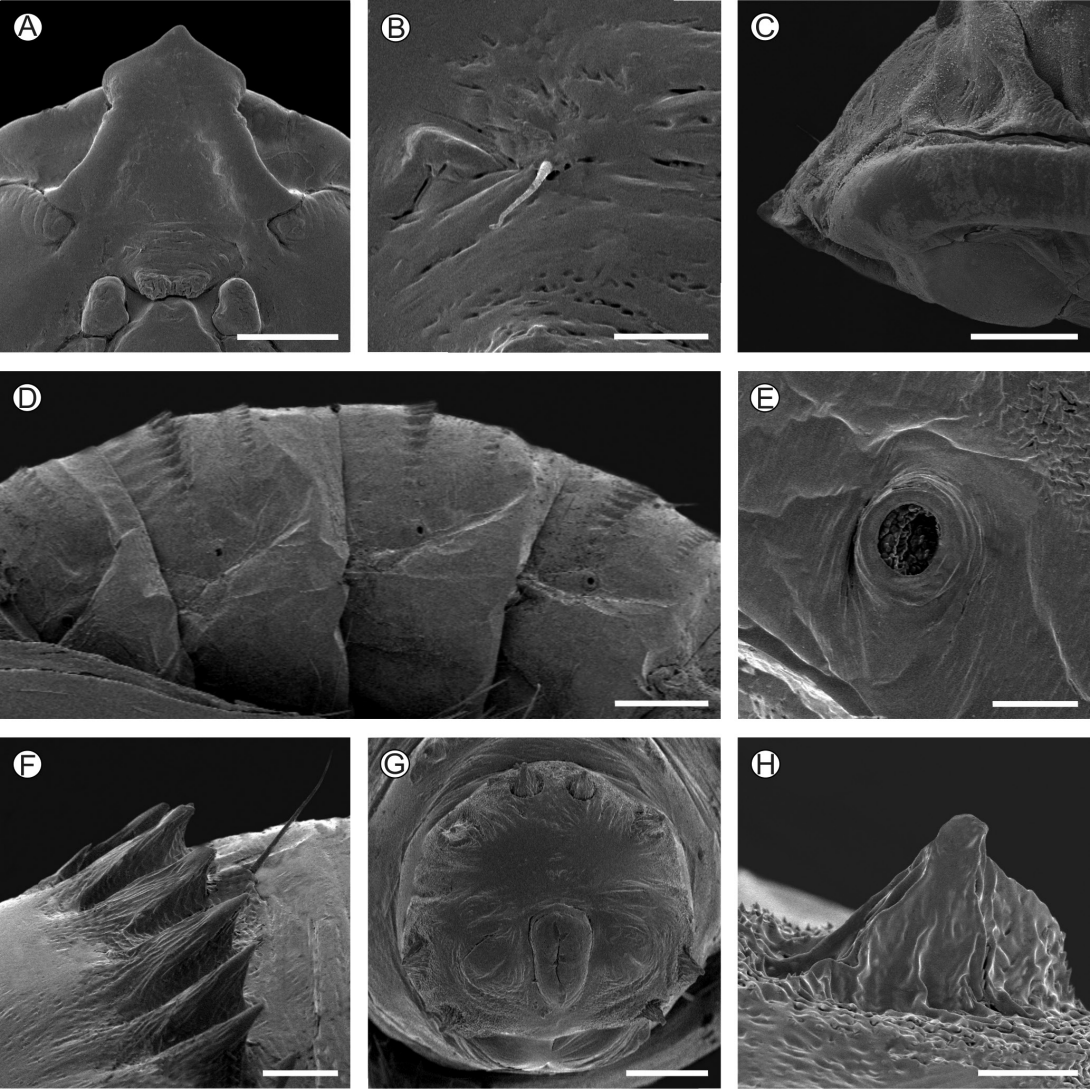
*Neospheciacecidogena* pupal morphology under scanning electron microscopy **A, C** head, ventral and lateral views, respectively **B** clipeal seta in detail **D** third to sixth abdominal segments, lateral **E** fourth abdominal spiracle, lateral **F** spines of fourth abdominal segment in detail, lateral **G** last abdominal segments, posterior **H** spine of last abdominal segment, mesal. Scale bars: 40 µm (**H**); 50 µm (**B, E**); 100 µm (**F**); 200 µm (**C, G**); 0.4 mm (**A**); 0.5 mm (**D**), respectively.

**Pupa** (Figs 7, 8). Body length (average ± standard deviation) = 11.52 ± 0.67 mm; *n* = 5. Yellowish brown, becoming dark brown near adult emergence (Fig. 7C). Head with stout, short, bow-shaped frontal gall-cutter process in dorsal view (Figs 7A, C), which is continued latero-caudally up to eye margin by slightly elevated ridges that limit depressions on frons under lateral view. Vertex with two pairs of small setae laterally. Clypeus little pronounced, with one pair of small setae laterally; labrum short, slightly bilobed (Fig. 8B). Antennae clubbed at the end, reaching anterior portion of third abdominal segment. Mandibles small, rounded, meso-anterior to the eyes. Maxillary palpi small, rounded, latero-posteriorly to the eyes. Proboscis shorter than and laterally margined by the prothoracic legs; galea converging mesally along the second half portion. Labial palpi contiguous on the center, extending to half length of the galea. Pronotum fairly developed, bearing a central ridge that extends caudally along the meso- and metanotum. Hindwings concealed by forewings, both extending to sixth abdominal segment. Protho-, meso-, and methatoracic legs reaching the second, fifth, and seventh abdominal segments, respectively. Thoracic and abdominal setae extremely reduced in size: one pair, latero-dorsally, on meso- and metathorax, and A2–A9; another pair, subspiracular, on A2–A7. Abdominal spiracles rounded, with slightly elevated peritreme (Fig. 8E), laterally on A2–A7; spiracle on A8 closed. Basal and caudal transverse rows of spines (Figs 8D, F) present from abdominal segments two to seven on males; only one row of such spines is found on segment seven of females, and also on segments eight and nine on both sexes. Last abdominal segment with four pairs of stout, scaly spines on caudal margin: two pairs in latero-dorsal and two pairs in latero-ventral position (Fig. 8H, G).

##### Distribution.

This new species is known only from the type locality, the humid forest portions of the CPCN Pró-Mata, São Francisco de Paula municipality, Rio Grande do Sul State, Brazil.

##### Host plant.

**Figure 9.**
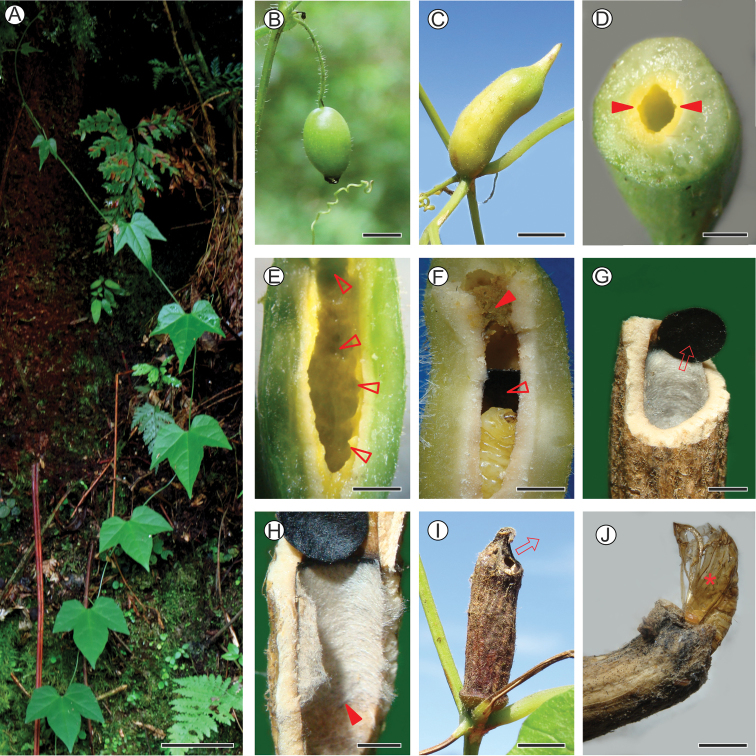
Natural history of *Neospheciacecidogena* on *Cayaponiapilosa***A** host plant at the type locality **B** young fruit, lateral **C** fully developed gall on axillary region, lateral **D, E** basal portion of median-sized gall sectioned transversally, showing yellowish nutritive tissue (pointed by closed arrows) (**D**) longitudinally sectioned medium-sized galls, showing larval feeding scars on nutritive tissue (some are indicated by open arrows) **F** longitudinally sectioned mature gall, with last instar larva (asterisk) inside (closed and open arrows indicate frass and operculum, respectively) **G** transversally sectioned, senescent gall showing detached operculum (indicated by seta) and internal wall covered by silk **H** longitudinally sectioned senescent gall showing internal silk covering (proximal limit pointed by closed arrow) **I** senescent, overwintering gall, lateral (seta indicates direction of adult emergence) **J** distal portion of senescent gall, showing pupal exuvium left partially protruded after adult emergence (marked with asterisk). Scale bars: 2 mm (**G, H**); 3mm (**D–F**); 4mm (**J**); 6 mm (**I**); 9 mm (**C**); 1 cm (**B**); 9 cm (**A**).

Galls of *N.cecidogena* have been found only in association with *Cayaponiapilosa* (Vell.) Cogn. (Cucurbitaceae), which is distributed in the ombrophilous Atlantic Forest of southern Brazil (from Minas Gerais to Rio Grande do Sul State) (Gomes-Klein et al. 2015). Biology and natural history of this cucurbit are poorly known. It is a herbaceous, prehensile vine (Fig. 9A), which bears pairs of forked, axillary tendrils, simple, alternate leaves with lamina that may vary from entire, to three to five lobed; flowers are solitary, axillary, and with penduncles varying from 7 to 9 cm long; fruits are ellipsoid and ca 2 cm in length, which are initially green (Fig. 9B) but changing to wine-colored when mature (Porto 1974; Villagra and Romaniuc Neto 2011). At the type locality, *C.pilosa* plants are found scattered on forest borders, particularly along trails.

##### Natural history.

The unilocular, cylindrical galls of *N.cecidogena* measure on average (± standard deviation) 3.44 ± 2.51 cm (*n* = 9) in length when mature. They appear individually and from the beginning develop externally on axillary buds of *C.pilosa* vines. Contrary to the oval *C.pilosa* fruits (Fig. 9B), *N.cecidogena* galls are not pedunculate (Fig. 9C). They are green during development and later turn dark brown with the progress of senescence (Fig. 9I). The internal chamber is filled with a yellowish nutritive tissue (Fig. 9D, E) which is consumed by larvae during development. With the end of feeding, the last larval instar builds a blackish, circular operculum (Fig. 9F–H) that splits the chamber into two sections, one distal, where the frass is deposited, and one basal, which has the distal portion of the wall lined with light-gray silk (Fig. 9E) and where pupation occurs. Achieving maturation, the wall of the gall hardens with the exception of the distal, pointed end, which remains thin and soft and through which adult emergence occurs (Fig. 9I). During emergence, with the action of the frontal process and body contortions, the pupa detaches the operculum and ruptures the distal, weaker portion of the wall. By continuing these movements and anchoring the body laterally with its abdominal spines, the pupa pushes itself partially out of the gall. During this process, the anterior portion of the exuviae is split, allowing adult emergence. In all cases of adult emergence under laboratory conditions, the anterior part of the pupal exuviae (head and thorax) was found protruding to the outside (Fig. 9J), while the posterior third remained in the chamber.

A few *C.pilosa* plants have been found at the type locality bearing from one to five *N.cecidogena* galls per plant. Field collections carried out during five consecutive years at the type locality indicated that it is a univoltine species, larvae growing during the summer when young galls are seen on *C.pilosa* vines. Fully developed galls containing last instar larvae have been collected mainly during autumn. When brought to the laboratory, these remained larvae during the winter, apparently in a diapause state. Pupation in this case occurred during the first week of September and emergence a few days later during early spring. The absence of a proboscis suggests that adults of *N.cecidogena* are not active feeders. The appearance of a substantial number of corionated eggs in the abdomen of dissected females shortly after emergence in the laboratory indicates that reproduction occurs early in adult life, and thus, adults may not live long.

## Discussion

A few molecular phylogenies have been proposed for Sesiidae, particularly including Melittini (McKern et al. 2008; Hans et al. 2012; Lait and Hebert 2018), which present a controversial relationship within Sesiinae, placed in the basal position. *Melittia* is the main representative of the tribe so far sequenced, and in this study presented high internal divergence (6–11%),which suggests that distinct lineages have been lumped into this genus. This seems to hold true particularly for the “New World” lineages that were separated from *Melittia* based on genitalia (Arita and Gorbunov 1996). Molecular data presented here gave further support in the sense that the genus *Neosphecia* is closely related to *Melittia*, from which COI sequences of *N.cecidogena* diverged ca 13%. Corresponding divergences between the genera *Synanthedon* and *Carmenta* were 18 and 22%, respectively. Unfortunately, DNA sequences are not available for the genera *Premelittia* and *Melittina*, which precludes further comparison within the Neotropical Melitiini, whose phylogenetic relationships are still controversial, as already mentioned.

*Premelittia* and *Melittina* are poorly known monotypic genera, with data available restricted to the original taxonomic description, and observations on morphology made *a posteriori* based also on the adult female holotypes only by Arita and Gorbunov (1996). According to these authors, the Nearctic and Neotropical Melittini as a whole are in need of revision, and thus, the taxonomic positioning of these groups within the family is still pending. Results presented in the present study demonstrate that variation among the immature stages of Sesiidae, especially the larva and its feeding habits, is broader than previously thought and should be taken into account in future phylogenetic studies involving these lineages. We demonstrate clearly that not only internal damage related to mobile (borer) activity is to be found among clearwing moths, but also typical, external galls containing differentiated nutritive tissues as induced by *N.cecidogena*.

Sesiid eggs have been described as ventrally flat, being laid with the longitudinal axis parallel to the substrate and having the lower surface less ornamented than the upper one (Duckworth and Eichlin 1974; Eichlin and Passoa 1983; Eichlin 1995; Puchi 2005). This may not the case in *N.cecidogena*, a situation that should be better examined under natural conditions. Eggs dissected in this study were transversally nearly elliptical and uniformly covered by a continuous plastron, with the exception of the anterior pole where the micropyles are located, as already mentioned. We could not identify differentiated aeropyles on the chorion surface of *N.cecidogena* eggs, which are supposedly located underneath such a plastron.

Unlike larvae of other sesiids that have pseudopodia bearing crochets (Heppner and Duckworth 1981; Heppner 1987), those of *N.cecidogena* lack such structures. It is still uncertain whether this reduction is unique to *Neosphecia* species. It may represent an evolutionary loss, associated with the stationary way of life within a gall. This hypothesis should be tested further by comparing this species with other representatives including those having similar cecidogenous habit (sessile, confined life style) where corresponding convergences to *N.cecidogena* is expected, and to different life habits like borers (more mobile life style), where pseudopodia bearing crochets are expected to be found. This could be also the case of setae that are reduced in size and in number, and which are tentatively named here. For example, P and Md groups are well developed in the head of other sesiids (e.g. Eichlin and Passoa 1983) but absent in *N.cecidogena*, except for P1. Heppner and Duckworth (1981) and Heppner (1987) stated that the L group is trisetose on the protorax of sesiids in general, which is not the case in *N.cecidogena* where this group is bisetose, also on abdominal segments A1–8. Furthermore, we could not find any trace of SV2 setae on *N.cecidogena*, except for the prothoracic segment; the SV3 and MV setae were not found.

Pupae of *N.cecidogena* showed the general integument morphology usually found in the family (Patočka and Turčani 2005). Pads of galea are well differentiated externally on *N.cecidogena* pupa, but contrary to other buccal appendages that are filled with the corresponding adult buccal appendages, they are empty later in pupal development, thus suggesting that the proboscis is in fact being lost during that ontogenetic stage in this species. Additional differences exist in the pupal stage of *N.cecidogena* in relation to the absence of a differentiated frontal process on the head, which is well developed and bow-shaped, for example, in *Melittiagilberti* Eichlin (Eichlin 1992). It also slightly differs regarding the shape, size, and arrangement of A10 spines, compared for example to those described for *Carmenta* species (Eichlin and Passoa 1983; Puchi 2005). Most sesiids pupate internally, within the gallery opened by larval feeding (e.g. Bruch 1941; Duckworth and Eichlin 1974; Eichlin 1995; Harms and Aiello 1995; Newland and Sawyer 2014). In this case, the larva may either tunnel to the plant surface prior to pupation, leaving a thin exterior covering through which emergence occurs later (Duckworth and Eichlin 1974), or build a cocoon within the feeding site that is surrounded by the frass (e.g. Lopes et al. 2003). However, other species leave the feeding site later in development, weaving a silk cocoon in the soil within which pupation occurs (e.g. Heppner and Duckworth 1981; Gorbunov 2015). In *N.cecidogena* pupation happens inside the gall, which is used as the pupal chamber after being closed distally with a silk-made operculum and deprived of frasses, and thus without tunneling or building a typical cocoon. Variation in this life history trait among other sesiid lineages should be also evaluated further from a phylogenetic perspective.

Finally, the galls described here appeared figured twice in association with *Cayaponia* spp. in a survey carried out at the type locality by Toma and Mendonça (2013). The one in early development stage that was illustrated by them (green color; Toma and Mendonça 2013: fig. 1P) had the induction attributed to an unidentified species of Coleoptera, which resulted from misidentification. The one that appears in a senescent stage (brown color; Toma and Mendonça 2013: fig. 1Q) had the induction correctly associated by these authors to an unidentified Lepidoptera. Similar galls are also found on *C.pilosa* plants in Parque Nacional do Itatiaia Nacional, Rio de Janeiro, as illustrated by Maia and Mascarenhas (2017: fig. 139). However, it is unlikely in this case that they are conspecific to *N.cecidogena* as the galls are differently shaped; they are pedunculate and present five conspicuous filiform projections distally. Bruch (1941) showed the feeding damage made by larvae of *Melittiatayuyana* Bruch, 1941, which appears externally as swellings on the stems of *Cayaponiaficifolia* (Vell.) Cogn. [= *C.bonariensis* (Mill.) Mart. Crov.] in Argentina, a plant that is sympatric to *C.pilosa* (Gomes-Klein et al. 2015). Possible tuberculate galls that are induced by an unidentified species of *Sincara* Walker, 1856 on the roots of *Wilbrandriaverticillata* (Vell.) Cogn. (Cucurbitaceae) were illustrated by Lima (1945), also for the Atlantic Forest in Brazil. Recently, Gorbunov (2015) suggested the existence of a gregarious feeding habit on galls induced by *Melittiaambo* Gorbunov, 2015 at the root collar of *Citrulluscolocynthis* (L.) Schrad. (Cucurbitaceae) in Ethiopia. These few records suggest cucurbit galls are associated with more than one lineage of sesiid moths, and may vary in general shape, host plant species, plant parts where they are induced, and the density of larvae they bear inside.

## Supplementary Material

XML Treatment for Melittina

XML Treatment for Premelittia

XML Treatment for Neosphecia

XML Treatment for Neosphecia cecidogena
